# Contents and quality of travel tips on malaria in English and Spanish travel blogs

**DOI:** 10.1186/s12936-021-03864-2

**Published:** 2021-08-16

**Authors:** Manuel Linares-Rufo, Laura Santos-Larrégola, Miguel Górgolas Hernández-de-Mora, José-Manuel Ramos-Rincón

**Affiliations:** 1 Microbiology Service, Hospital Universitario Príncipe de Asturias, Alcalá de Henares, Madrid Spain; 2 Foundation iO, Madrid, Spain; 3 Buenos Aires Health Centre, Madrid, Spain; 4 Department of Medicine, Universidad Autónoma de Madrid, Madrid, Spain; 5 Department of Medicine, Universidad Miguel Hernández de Elche, Alicante, Spain

**Keywords:** Malaria, Traveler, Tourist, Blog, Social Networks, Traveler Advice, Chemoprophylaxis, Prevention

## Abstract

**Background:**

Europe has about 10,000 imported cases of malaria each year, or around 80 cases per 100,000 trips to endemic areas. Non-use of chemoprophylaxis in travellers remains the main reason for this. The proliferation of online travel blogs as a source of advice (sometimes the only one used) for preparing a trip to an endemic area may play a role in the decision to use chemoprophylaxis. The aim of this study was to analyse the information offered on malaria in the main travel blogs in English and Spanish.

**Methods:**

Five hundred travel blogs in English and 100 in Spanish, considered highly relevant were analysed. The relevance were according to different metrics: (1) Alexa Rank; (2) social networks (RRSS) measuring the total followers of Facebook, Twitter, Instagram and YouTube; (3) number of monthly visits using the SEMrush tool; (4) domain authority; and (5) number of backlinks or incoming links using the SEMrush tool.

**Results:**

Of the included travel blogs, 57% of those in English and 64% of those in Spanish offered information on malaria, and 79 and 75%, respectively, featured a discussion on malaria written as a blog post or in forum comments. Information on chemoprophylaxis was available in 56.1% of English-language blogs and 10.7% of Spanish-speaking blogs, while its side effects were discussed in 38.6 and 68.8%, respectively (p < 0.001). Content analysis revealed that the information was usually insufficient, incomplete or, more seriously, inaccurate. In many cases, this could discourage users from taking appropriate preventive measures.

**Conclusions:**

Travel blogs in English and Spanish provide low-quality information on malaria. The so-called “travel influencers” must communicate reliable, verified and quality information on malaria on their channels in a way that could contribute to reducing the burden of the disease in travellers.

**Supplementary Information:**

The online version contains supplementary material available at 10.1186/s12936-021-03864-2.

## Background

About 10,000 imported cases of malaria are reported in Europe each year, or about 80 for every 100,000 trips to endemic areas. The global incidence of the disease was estimated at 229,000,000 cases in 2019, of which more than 50% occurred in Africa [1]. Frequent international travel combined with the inadequate use of preventive measures in travellers result in a high number of imported malaria cases worldwide [2, 3].

Despite the existence of official databases, the real burden of imported malaria in non-endemic countries is probably underestimated [4]. Prevalence estimates are subject to important limitations, as only surveys are available rather than the actual number of travellers by origin and destination. In the USA, the Centers for Disease Control and Prevention (CDC) reported 2161 confirmed imported malaria cases with onset of symptoms in 2017. In the European Union, the European Centre for Disease Prevention and Control (ECDC) reported 8641 malaria cases in 2019 [5]. Other countries such as Canada, Australia or the UK report quite similar numbers of cases per 1 million population [6]. In 2019, tourist travel increased in all regions of the world by 3.8% over the previous year, reaching 1.5 billion travellers globally. Asia-Pacific saw growth of 4.6% and Africa, 4.2%, higher than the global average [7].

The risk of imported malaria is known to be higher in travellers to Africa, especially migrants and people visiting friends and relatives (VFRs). There are many studies that indicate that chemoprophylaxis use is still insufficient in people with imported malaria cases, both in these high-risk groups as well as young people and travellers with a high educational level [8, 9].

The first source of information that the traveller can access is the official, “classic” route, that is, specific, timely advice offered by health professionals through specialized clinics [10]. However, informal sources should not be overlooked, as they complement professional advice and condition travellers’ choices and attitudes with regard to how to act when travelling to a malaria-endemic country. Travel blogs in particular have proliferated across the web, offering a firsthand, layperson’s account on different destinations. Thus, the objective of this study was to analyse the information offered on malaria in the main travel blogs in English and Spanish.

## Methods

Six hundred travel blogs (500 in English and 100 in Spanish) with general content for travellers and accounts of trips to malaria-endemic countries were analysed. The blogs were selected based on different metrics assessing their relevance (Additional file; Table S1, Table S2 1), using the following external tools: (1) Alexa Rank (a Global Search Engine Optimization [SEO] positioning system; Amazon Company, USA; https://www.alexa.com/siteinfo); (2) social networks (RRSS) measuring the total followers of Facebook, Twitter, Instagram and YouTube), using Influencer Marketing Hub (https://influencermarketinghub.com/), Fanpage Karma (https://www.fanpagekarma.com/) and Metricool (https://metricool.com); (3) number of monthly visits using the SEMrush tool (https://es.semrush.com/); (4) domain authority, using the MOZ tool (https://moz.com/free-seo-tools); and (5) the number of backlinks or incoming links using the SEMrush tool. Moreover, the monthly and annual visits/users for all the English and Spanish blogs selected using the tools available on the Alexa website were estimated.

The blog’s own search engine was used to find content (text, video, or podcasts) that included the word “*malaria*”. The quality of the information was examined according to the 10 yes/no questions shown in Table 1.

**Table 1:** Quality items from Spanish- and English-language travel blogs

Quality items	English		Spanish		p value
	n/N	%	n/N	%	
Does the blog offer information on malaria?	285/500	57	64/100	64%	0.19
Is there a discussion on malaria in the forum or comments section?	225/285	79	48/64	75	0.49
Does the blog contain information on the side effects of chemoprophylaxis?	110/285	38.6	44/64	68.8	**< 0.001**
Is the information provided up-to-date?^a^	60/285	21.1	22/64	34.4	**0.023**
Does the blog offer links to official/specialized travel medicine websites?	45/285	15.8	10/64	15.6	> 0.99
Does the blog advise non-certified preventive products against malaria or whose effectiveness is questionable?	15/285	5.3	10/64	15.6	**0.007**
Does the blog have adequate information on chemoprophylaxis?	160/285	56.1	7/64	10.9	**< 0.001**
Is the information that the blog provides referenced?	35/285	12.3	4/64	6.3	0.12
Does the blog provide correct information on standby emergency treatment?	20/285	7.0	2/64	3.1	0.28
Was the information on malaria generated or reviewed by a healthcare professional?	10/285	3.5	2/64	3.1	> 0.99

^a^Reviewed in the last year. In bold, statistically significant differences

The differences between English- and Spanish-language blogs were determined using Pearson’s chi-square test for categorical variables. Values were considered to be statistically significant when the p value was less than 0.05. Statistical data analysis was performed using IBM SPSS Statistics for Windows, Version 25.0 (Armonk, NY: IBM Corp).

## Results

Web traffic measurement tools show that collectively, the 600 blogs analysed have a reach of 92,400,000 unique visitors/month, for an incredible 1,108,800,000 visitors per year. No information on malaria was available in 43% of the 500 English-language or in 36% of the 100 Spanish-language blogs, even though they were pages that offered recommendations for visiting endemic countries. On the other hand, about 95% of Spanish blogs did offer advertising or advertising content for travel health insurance.

Of the blogs that contained information on malaria (English n = 285 and Spanish n = 64), 79 and 75%, respectively, mentioned malaria in the forum or comments section. Information on chemoprophylaxis was available in 56.1% of English-language blogs and 10.7% of Spanish-speaking blogs, while its side effects were discussed in 38.6 and 68.8%, respectively (p < 0.001). Only 21.1 and 34.4% (p = 0.023) of the blog posts presented up-to-date information (reviewed in the previous year) (Table 1).

Only 15.8% of the English- and 15.6% of the Spanish-language blogs provided links to official/specialized websites (e.g. international vaccination centers, Ministry of Health, other specialized websites dedicated to travel medicine). A minority (English 5.3%, Spanish 15.6%, p = 0.007) advised uncertified preventive products against malaria or products of dubious effectiveness, such as garlic, onion, vitamin B, bracelets, nitrous oxide, various cosmetics, anti-mosquito candles, or electrical/ultrasonic devices to repel mosquitoes. Few blogs (English 12.3%, Spanish 6.3%) included references for the information presented. Only 7.0 and 3.1% of the blogs, respectively, provided correct information about self-treatment, while healthcare professionals generated or reviewed the information in just 3.5 and 3.1% of the blogs (Table 1).

In the qualitative analysis uncovered several important misconceptions in the blogs (Table 2), reflecting the main pitfalls found in the analysis of malaria chemoprophylaxis. Some key points have been detected which could inform interventions to improve advice.

**Table 2:** Patterns of misconceptions around chemoprophylaxis for malaria, as identified in qualitative analysis of travel blogs

English-language blog
Although the information offered by the authors on chemoprophylaxis was correct, topics generated by visitors often contained incorrect advice or comments that could discourage the use of antimalarial prophylaxis. Frequently, these comments were not refuted by the blog authors, perhaps due to their fear of losing followers
The posts address the financial cost of chemoprophylaxis pills for the traveller, quantifying these from a cost-benefit point of view but without a scientific basis
It is common to find numerous self-referenced links to the website itself on various aspects of malaria (risk areas, pregnancy, use of repellents …) instead of links to official health websites. It seems that more value is placed on attracting traffic to the site (SEO) than providing accurate or serious travel advice, or perhaps bloggers seek to avoid projecting an image that is too serious
Bloggers often make subjective comments with a negative or neutral tone about chemoprophylaxis, for example: “If there is no other choice, we will take it,” “We have not seen a mosquito the whole trip,” “In Nepal we found a traveller taking chemoprophylaxis at 2800 meters, what crazy doctor advised her?” This type of comment can generate or contribute to reluctance to start any prophylaxis
Spanish-language blogs
Twelve recommended “*the malaria vaccine*” for travel, showing clear confusion with the concept of immunization versus chemoprophylaxis.
The dosage of antimalarials in many cases was wrong or their indication was outdated (for example, they spoke of using chloroquine in areas of current resistance).
The side effects of chemoprophylaxis were described in 44 blogs, described in language such as: “*poison,” “serious complications,” “aggressive pills,” or “psychotic attack.*”
Some of the blogs recommended products based on vitamin B or repellants and devices against mosquitoes of dubious or scientifically unproven efficacy.

## Discussion

This study showed the low quality of information about malaria published on travel blogs in English and Spanish. The content was generally insufficient, incomplete or, worse, inaccurate, with the result being that the users may be discouraged from taking adequate preventive measures. The scientific quality of these blogs may be related to a greater number and severity of imported malaria cases, as travellers may follow advice without an adequate scientific basis when making decisions about malaria prevention.

Efforts in malaria prevention in other environments, such as travel agents or specific groups of travellers, are well known [11]. However, to date these initiatives have been practically nonexistent in the field of travel blogs, despite the current context of “infoxication” or online fake news [12].

Sometimes travellers’ perceptions of malaria risks are unrealistic. Travel blogs should thus correct misperceptions (such as believing that curing malaria is easier than taking prophylaxis or that travellers visiting relatives have some level of innate immunity). Certain “filter criteria” could also be applied, allowing the user to evaluate the information obtained for themselves. However, in other, so-called “mainstream” media, such as newspapers, radio or television, the information presented may also be erroneous, incomplete or biased, but that is not cause for it to be withdrawn. Rather, the user must apply their own personal judgment.

The adoption of codes of conduct and ethics, such as HONcode [13], could be a more reliable way to favour the generation and dissemination of high-quality, accurate information. Since a significant proportion of people have low health literacy and difficulties accessing information, many audiences may find it challenging to assess the quality of the information and apply it to their own circumstances [14]. The so-called “travel influencers” must, therefore, communicate reliable, verified and high-quality information on malaria in a way that could contribute to reducing the burden of disease in travellers.

Introducing a code of conduct for sensitive information provided by these sites is further justified by the fact that malaria prevention is just a small drop in the ocean of popular medical communication. Indeed, the larger problem is so huge that it is really hard addressing this single aspect alone, or even medical travel advice in general. Good initiatives exist, including the HONcode, but most people are not aware of them [15]. Moreover, many prestigious medical and academic sites do not adhere to HONcode, which can by no means be enforced. However, this work is a first step in an open and relevant line of work [16].

The main strength of the study resides in the fact that it opens the door to innovative interventions to improve malaria prophylaxis. It also touches on a much wider topic of interest than the specific focus of the study, namely the ability to refer blog readers to quality information to improve the training of travellers.

However, the results of this study should be interpreted in light of the study’s limitations. First of all, the study was performed in only English and Spanish blogs, which may be different from blogs in other languages. Secondly, the study did not differentiate between the country where the bloggers were based. Third, the criteria used for measuring quality was based on researchers’ subjective experience.

In conclusion, travel blogs written in English and Spanish often provide low-quality information on malaria. The contents offered were generally insufficient, incomplete or, worse, inaccurate. Further qualitative studies are necessary to analyse the quality of information on malaria in online media.

## Supplementary Information

**Additional file 1: Table S1.** 500 English travel blogs analyzed by alphabetic order. **Table S2.** 100 Spanish travel blogs analyzed by alphabetic order.

## Data Availability

J.M.R.R. has full access to and is the guarantor for the data. The datasets generated are available from the corresponding author on reasonable request.
