# Supplementary material for: Contents and quality of travel tips on malaria in English and Spanish travel blogs
Source: Malar J. 2021 Aug 16;20:342. doi: 10.1186/s12936-021-03864-2 (PMC8365983; doi:10.1186/s12936-021-03864-2)
Supplement: Supplementary file 1 — Additional file 1: Table S1. 500 English travel blogs analyzed by alphabetic order. Table S2. 100 Spanish travel blogs analyzed by alphabetic order. [file 12936_2021_3864_MOESM1_ESM.docx]

Additional material.

**Table S1. 500 English travel blogs analyzed by alphabetic order**

| 1 | [1 Dad, 1 Kid – One Crazy Adventure. Giving my Kid the World.](http://1dad1kid.com/) |
| --- | --- |
| 2 | [1 Fun Girl Travels – A Penchant for the Offbeat](http://1fungrltravels.com/) |
| 3 | [A Backpacker’s Tale – Hitting the Road Bumps so You Don’t Have To](http://www.abackpackerstale.com/) |
| 4 | [A Brit and A Southerner – Explore the World One Weekend at the Time](http://abritandasoutherner.com/) |
| 5 | [A Broken Backpack – Budget Backpacking & Long Term Travel](http://abrokenbackpack.com/) |
| 6 | A Couple For The Road – Cultural Travel Blog |
| 7 | [A Dangerous Business – One Ordinary Girl, Life’s Extraordinary Adventures](http://www.dangerous-business.com/) |
| 8 | [A Feeder Travels – Food Fueled Adventures Around the Globe](http://www.afeedertravels.com/) |
| 9 | [A Jaunt with Joy – Outdoor Tips, Wildlife Conservation and Getting Into Nature](http://joyandjourney.com/) |
| 10 | [A Little Adrift – Journey Toward Knowledge & Perspective](http://alittleadrift.com/) |
| 11 | [A Nerd at Large – The Geektastic Travel Blog](http://www.anerdatlarge.com/) |
| 12 | [A Speck In Time – With A Curious Mind & Wandering Heart](http://www.aspeckintime.com/) |
| 13 | [A Wanderlust For Life – Expat and European Travel Blog](http://www.awanderlustforlife.com/) |
| 14 | [A World to Travel – Unique Travel Experiences](http://www.aworldtotravel.com/) |
| 15 | [Absolutely Lucy – Adventure Seeker, From Backpacking to Luxury Treats](http://www.absolutelylucy.com/) |
| 16 | [Active Planet Travels – Traveling the World](http://activeplanettravels.com/) |
| 17 | [Adventure Mom – Adventure Travel and Lifestyle](http://adventure-mom.com/) |
| 18 | [Adventures Around Asia – Travel & Expat Lifestyle Blog](http://adventuresaroundasia.com/) |
| 19 | [Adventurous Kate – Solo Female Travel Blog](http://adventurouskate.com/) |
| 20 | [Afaranwide – Married couple, with experience as expats, travellers and tourists.](http://afaranwide.com/) |
| 21 | [Against The Compass – Off The Beaten Path Travel Blog](https://againstthecompass.com/en/) |
| 22 | [Alexandra Luella – For the Love of Writing and Story Hunting](http://alexandraluella.com/) |
| 23 | [Alison’s Adventures – Your Passport To The World](http://alisonsadventures.com/) |
| 24 | [Along Dusty Roads](https://www.alongdustyroads.com/) |
| 25 | [Always Wanderlust – Eat, Sleep, Travel, Repeat](https://alwayswanderlust.com/) |
| 26 | [Am I Nearly There Yet – Travel Inspiration, Photography, and Advice](http://aminearlythereyet.com/) |
| 27 | [Amateur Traveler – The Best Places to Travel](http://amateurtraveler.com/) |
| 28 | [American Travel Blogger – An American Exploring the World](http://www.americantravelblogger.com/) |
| 29 | [Angie Away – Travel the World with the Girl Next Door](http://angieaway.com/) |
| 30 | [Anna Everywhere – Chic Adventure Travel Blog. Travel with Purpose](http://annaeverywhere.com/) |
| 31 | [Anxious & Abroad – Travel Guide for the Anxious Backpacker](https://www.anxiousandabroad.com/) |
| 32 | [Anywhere We Roam](https://anywhereweroam.com/) |
| 33 | [Around the World “L” – Learning & Loving Global Education](http://www.aroundtheworldl.com/) |
| 34 | [Around the World in Katy Days – Environmentalist Adventuring on a Budget](http://aroundtheworldinkatydays.com/) |
| 35 | [Art of Non-Conformity – Unconventional Strategies for Life](http://chrisguillebeau.com/) |
| 36 | [Atlas & Boots](https://www.atlasandboots.com/) |
| 37 | [Aussie on the Road – In Search of a Life Less Ordinary](http://www.aussieontheroad.com/) |
| 38 | [Autumn Aquarius Adventures – Adventures and Dreams of a Girl that Travels](https://jamiebethlaird.com/) |
| 39 | [Away Go We – Inspiration from less-developed countries](https://www.awaygowe.com/) |
| 40 | [Backpack ME – India meets Portugal & Sets Off to Travel the World](http://bkpk.me/) |
| 41 | [Backpacking Africa For Beginners – Traveling In Africa For the First Time](http://backpackingafricaforbeginners.com/) |
| 42 | [Backpacking Man – Adventure Solo Male Travel Blog](http://backpackingman.com/) |
| 43 | [Backpacking Matt – Budget Travel Advice, Inspiration, & Ideas](http://www.backpackingmatt.com/) |
| 44 | [Bacon is Magic – Culinary Travel Sit About the Best Food Around the World](https://www.baconismagic.ca/) |
| 45 | [BBQ Boy – Travels of BBQ Boy & Spanky](http://bbqboy.net/) |
| 46 | [Be My Travel Muse – Off-Beat Solo Female Adventure Travel](http://bemytravelmuse.com/) |
| 47 | [Be on the Road – Travel & Photography Blog](http://www.beontheroad.com/) |
| 48 | [Bearfoot Theory – Exploring the Best in the West](http://bearfoottheory.com/) |
| 49 | [Become Nomad – Long Term Travel & Digital Nomad Lifestyle](http://becomenomad.com/) |
| 50 | [Been Around The Globe – What It’s Like To Travel As A Black Person](https://www.beenaroundtheglobe.com/) |
| 51 | [Bel Around the World – Travel Resources & Inspiration for Millennial Travelers](http://belaroundtheworld.com/) |
| 52 | [Belize Adventure Travel – Travel in Belize](http://www.belizeadventure.ca/) |
| 53 | [Blissful Guro – Adventures Of A Public School Teacher](http://www.blissfulguro.com/) |
| 54 | [Blue Sky Traveler – GenX Travel Blog](http://www.blueskytraveler.com/) |
| 55 | [Bohemian Trails – Global Art & Culture for the Avant-Garde Traveler](http://www.bohemiantrails.com/) |
| 56 | [Borders of Adventure – Destination Inspiration with a Social & Cultural Twist](http://www.bordersofadventure.com/) |
| 57 | [Breathe Dream Go – India Oriented](https://breathedreamgo.com/) |
| 58 | [Breathe With Us – Cultural & Nature Travel Adventures](http://breathewithus.com/) |
| 59 | [Breathing Travel – Travel more & take your career on the road](http://breathingtravel.com/) |
| 60 | [Bren On The Road – Travel More, Spend Less, Enjoy Life](http://www.brenontheroad.com/) |
| 61 | [Bridges & Balloons – Digital Nomad Tales, Tips and Inspiration](http://www.bridgesandballoons.com/) |
| 62 | [Brit on the Move – See and try everything – while maintaining a day job!](http://britonthemove.com/) |
| 63 | [Bucket List Publications – Travel, Adventure, and New Experiences](https://lesleycarter.wordpress.com/) |
| 64 | [Bucketlistly Travel Blog](https://www.bucketlistly.blog/) |
| 65 | [Bunch Of Backpackers – Extraordinary Adventures and Travel Advice](https://www.bunchofbackpackers.com/) |
| 66 | [Camels & Chocolate – Female Tales from a Travel Addict](http://camelsandchocolate.com/) |
| 67 | [Candice Does the World – Personal Stories of Misadventure](http://www.candicedoestheworld.com/) |
| 68 | [Captain & Clark – Adventurous Lovebirds with Severe Cases of Wanderlust](http://captainandclark.com/) |
| 69 | [Caroline in the City  – Escaping the Expectations of the ‘Real World’](http://www.carolineinthecityblog.com/) |
| 70 | [Castaway with Crystal – Alternative Lifestyle with Budget Green Travel](http://castawaywithcrystal.com/) |
| 71 | [Celebrate the Weekend – Boston-Based Family Travel & Leisure Blog](http://celebratetheweekend.com/) |
| 72 | [Changes in Longitude – Field-Tested Travel Tips](http://www.changesinlongitude.com/) |
| 73 | [Charlie on Travel – Sustainable Slow Travel](http://charlieontravel.com/) |
| 74 | [Chasing Coconuts – Travel Blogger from Auckland New Zealand](http://chasingcoconuts.co/) |
| 75 | [Chasing the Unexpected – The World Beyond the Guidebook](http://www.chasingtheunexpected.com/) |
| 76 | [Chronicles In Wonderland – Tales Of Traveling](http://chroniclesinwonderland.com/) |
| 77 | [Ckanani – Luxury Travel & Adventure Blog](https://ckanani.com/) |
| 78 | [Claire’s Itchy Feet – Solo Female Travel Blog](http://www.clairesitchyfeet.com/) |
| 79 | [Couple’s Coordinates – Couples Travel Blog](http://couplescoordinates.com/) |
| 80 | [Curb Free with Cory Lee – The World from a Wheelchair User’s Perspective](http://curbfreewithcorylee.com/) |
| 81 | [David’s Been Here – Leave Your Mark on the World](http://davidsbeenhere.com/) |
| 82 | [Destinations Unkown – Dark tourism, wildlife, nature and UNESCO sites.](http://destinationsunknown.com/) |
| 83 | [Dish Our Town – Traveling the World, One Dish at a Time](http://dishourtown.com/) |
| 84 | [Ditch the Map – American couple who sold everything to travel the world](https://www.ditchthemap.com/) |
| 85 | [Don’t Forget To Move – Travel Deeper For Cheaper](http://dontforgettomove.com/) |
| 86 | [Dream Big Travel Far – For couple backpackers and type 1 Diabetics](http://www.dreambigtravelfarblog.com/) |
| 87 | [Drifter Planet – A Backpacker Couple](http://drifterplanet.com/) |
| 88 | [Drink Tea Travel – Canadians On A Sustainable Lifestyle Mission](http://www.drinkteatravel.com/) |
| 89 | [Dutch Dutch Goose – Family Friendly Travel in Europe](http://www.dutchdutchgoose.com/) |
| 90 | [Earth’s Attractions – Travel guides, tips and news](http://www.earthsattractions.com/) |
| 91 | [Eat Like a Girl – Food From Around the World by a Solo Female](http://eatlikeagirl.com/) |
| 92 | [Embolden Adventures – Encouraging Travel Around the World](http://www.emboldenadventures.com/) |
| 93 | [Emelie’s Travels – Adventures, Travel Stories and Photography](http://emeliestravels.com/) |
| 94 | [Erika’s Travels – Budget Blog Covering Off-the-Beaten Path Destinations](http://www.erikastravels.com/) |
| 95 | [Escaping Abroad – Escaped a Busy U.S. lifestyle to Travel the World](http://www.escapingabroad.com/) |
| 96 | [Escaping Reality – Escaping To Travel More](https://escapingreality.co.uk/) |
| 97 | [eTramping – Traveling the World on $25 a Day](http://etramping.com/) |
| 98 | [Etsy Explores – Slow Travel and Off the Path Adventures](http://www.etsyexplores.com/) |
| 99 | [Euriental – Luxury Travel & Style](http://euriental.com/) |
| 100 | [European Diaries  – Exploring Cultures, Places, Food and History in Europe](http://www.europediaries.com/) |
| 101 | [Event Traveller – Featuring Events](http://www.event-traveller.com/) |
| 102 | [Every Steph – Green & Glamorous Travel & Lifestyle](http://www.everysteph.com/) |
| 103 | [Everything Everywhere – Exploring the World’s UNESCO Sites](https://everything-everywhere.com/) |
| 104 | [Everything Zany – Photography & Lifestyle of Dual Citizen](https://everythingzany.com/) |
| 105 | [Explore With Erin – Traveling the World with Kids](https://explorewitherin.com/) |
| 106 | [Explorista – Female Travel. Digital Nomad](http://explorista.net/) |
| 107 | [Exsplore – Best Camping and Hiking Trips](https://www.exsplore.com/) |
| 108 | [Eye & Pen – Inspired Travel](http://www.eyeandpen.com/) |
| 109 | [Fab Meets World – UK Travel & Food Blogger](http://fabmeetsworld.com/) |
| 110 | [Faith’s Travels – Budget Travel With a Mom and a Down Syndrome Sister](http://www.faithstravels.com/) |
| 111 | [Feet Do Travel – Scuba Diving, Wildlife & Photography](http://www.feetdotravel.com/) |
| 112 | [Feet on the Map – Focus on Himalayas, Offbeat Journeys, Responsible Travels](https://feetonthemap.com/) |
| 113 | [Fernweh Sarah – Unspoiled beaches, luxury hotels & experiences](https://www.fernwehsarah.com/) |
| 114 | [Ferreting Out the Fun – Adventurous Spirit. Open Mind. More Fun.](http://www.ferretingoutthefun.com/) |
| 115 | [Finding the Universe – Travel Tales, Photography, & Humor](https://www.findingtheuniverse.com/) |
| 116 | [Five Adventurers – A Family of 5, Who Love to Explore the World](http://www.fiveadventurers.com/) |
| 117 | [Flashpacker Family – Traveling with the Family of 4](http://flashpackerfamily.com/) |
| 118 | [Fleeting Life – Lifestyle Hacks & Travel Deals](http://www.fleetinglife.com/) |
| 119 | [Floating My Boat – Solo Female Outdoors & Photography](http://floatingmyboat.com/) |
| 120 | [Fluent in 3 Months – Travel & Learn Languages](http://www.fluentin3months.com/) |
| 121 | [Food Travelist – Ambassadors of World Food Tourism](http://foodtravelist.com/) |
| 122 | [Footloose Dev – All about budget and adventure travel experiences](http://footloosedev.com/) |
| 123 | [For Two, Please – A Blog for Food Lovers and Adventure Seekers](http://fortwoplz.com/) |
| 124 | [Former Tourist – Engaging Travel Folks](http://www.formertourist.com/) |
| 125 | [Fotostrasse – Travel Blog by Berlin based Brazilian Couple](http://fotostrasse.com/) |
| 126 | [From Ice to Spice – Tales of Two Icelanders](http://fromicetospice.com/) |
| 127 | [Gaijin Crew – A Minimalist Travel Blog](https://gaijincrew.com/) |
| 128 | [Gallop Around the Globe – Independent and Cultural Exploration on a Budget](http://galloparoundtheglobe.com/) |
| 129 | [Gap Year Escape – Inspiration to escape the ordinary](http://gapyearescape.com/) |
| 130 | [Geeky Explorer – Travel Geek Sharing The Best Value](http://geekyexplorer.com/) |
| 131 | [Getting Stamped – Figuring Out Life One Stamp at a Time](http://www.gettingstamped.com/) |
| 132 | [Getting to Nowhere – Adventure Cycling Around the World](http://gettingnowhere.net/) |
| 133 | [Girl Eat World – An Adventure in Food & Travel](https://girleatworld.net/) |
| 134 | [Girl Gone Travel – Travel Inspiration for Friends, Family, and Lovers.](http://girlgonetravel.com/) |
| 135 | [Girl vs Globe – Stylish female travel and lifestyle](http://www.girlvsglobe.com/) |
| 136 | [Give into Adventure – Mountains, Hiking, Outdoor Adventure](http://www.giveintoadventure.com/) |
| 137 | [Global Grasshopper – Travel Photography, Tips and Inspiration](http://www.globalgrasshopper.com/) |
| 138 | [Globetrotter Avenue – Travel Stories from Around the World](http://www.globetrotteravenue.com/en/) |
| 139 | [Globetrotter Guru – Solo Female Traveller, Helping You to Earn Online](http://www.globetrotterguru.com/) |
| 140 | [Globetrotting Mama – See the World. Build A Life. Bring Your Family.](http://globetrottingmama.com/) |
| 141 | [Globo Treks – It’s all out there. Step outside.](http://www.globotreks.com/) |
| 142 | [Go Backpacking – Around the World Travel Blog](http://gobackpacking.com/) |
| 143 | [Go See Write – Overland Travel Adventures](http://goseewrite.com/) |
| 144 | [Go – The Travel Podcast](https://www.gothepodcast.com/) |
| 145 | [Goats on the Road – Turning Travel Into Lifestyle](http://goatsontheroad.com/) |
| 146 | [GoFam Travel – Traveling With Kids](https://gofamtravel.com/) |
| 147 | [Gomad Nomad – Travel Mag for Independent Travelers](http://gomadnomad.com/) |
| 148 | [Gone Sun Where – Part Time Londoner, Part Time Tourist](http://www.gonesunwhere.com/) |
| 149 | [Green Global Travel – Ecotourism & Nature/Wildlife Conservation](http://greenglobaltravel.com/) |
| 150 | [Grown-Up Travel Guide – Helping You Change a Backpack for a Suitcase](http://grownuptravelguide.com/) |
| 151 | [Grrrl Traveler – Imperfect Adventures of a Solo Traveler](http://grrrltraveler.com/) |
| 152 | [Half Half Travel – Travel With Becca & Dan](https://www.halfhalftravel.com/) |
| 153 | [Half This World Away – Luxury Travel Blog](http://www.halfthisworldaway.com/) |
| 154 | [Happy Frog Travels – Don’t Be A Tourist, Be A Traveller](http://happyfrogtravels.com/) |
| 155 | [Happy to Wander – Practical Advice on Balancing Travel with Real Life](http://happytowander.com/) |
| 156 | [Haute Fashion – Travel Blog About Textiles, Tribes and Traditional Dress](http://hauteculturefashion.com/) |
| 157 | [Heart My Backpack – Backpacking Off the Beaten Path](http://www.heartmybackpack.com/) |
| 158 | [Heart of Everywhere – Your Guide to Discover the Portuguese Hidden Gems](http://www.heartofeverywhere.com/) |
| 159 | [Heather On Her Travels – Travel Inspiration & Destination Information](http://heatheronhertravels.com/) |
| 160 | [Hecktic Travels – No Possessions. No Plans. Just Travel.](http://www.hecktictravels.com/) |
| 161 | [Helen in Wonderlust – Life is an Adventure. Live It!](http://www.heleninwonderlust.co.uk/) |
| 162 | [Her Coffee Diaries – Nw Blog About Budget Travel As a Student](http://hercoffeediaries.com/) |
| 163 | [Het Is De Merckx – A travel blog born out of the passion to make people laugh](http://hetisdemerckx.be/en/) |
| 164 | [Hippie in Heels – A Glamorous Travel Blog Mostly About India](http://hippie-inheels.com/) |
| 165 | [Hole in the Donut – Epic Adventures at an Affordable Price](http://holeinthedonut.com/) |
| 166 | [HollyDayz -Travel Experiences, Tips, Food, Reviews](http://hollydayz.com/) |
| 167 | [HoneyTrek – World’s Longest Honeymoon](http://www.honeytrek.com/) |
| 168 | [Hot Mama Travel – Family Travel Adventures](http://hotmamatravel.com/) |
| 169 | [I Am Aileen – Lifestyle & Travel Blog](https://iamaileen.com/) |
| 170 | [I Luv 2 Glove Trot – A Travel Site Focusing on Stories of Trotting the Globe](http://iluv2globetrot.com/) |
| 171 | [I Share These – Offbeat Locations, Food and Culture from India](http://isharethese.com/) |
| 172 | [I Should Log Off – Family Travels](http://ishouldlogoff.com/) |
| 173 | [In Due Time – Travel, Fertility, Fitness](http://www.in-due-time.com/) |
| 174 | [In My Suitcase – Travelling As a Freelance Digital Nomad](http://www.sabrinabarbante.com/) |
| 175 | [India Travel Blog – Travel Bloggers from India](http://www.indiatravelblog.com/) |
| 176 | [Indie Traveller – Inspiration & Honest Advice for Independent Travellers](http://www.indietraveller.co/) |
| 177 | [Infinite Juice – Driving Along the Pan-American Highway](http://www.infinitejuice.com/) |
| 178 | [Inside Our Suitcase – Maximise your travels regardless of your budget](http://insideoursuitcase.com/) |
| 179 | [Inside Out With Rahul Yuvi – About Travel & Life](https://insideoutwithrahulyuvi.com/) |
| 180 | [Inside the Travel Lab – Unusual Journey & Luxury](http://www.insidethetravellab.com/) |
| 181 | [Interlude Journey – Blogzine for Tips and Itineraries](http://interludejourney.com/) |
| 182 | [Island Girl in-Transit – Travel Blog Specializing in Caribbean Destinations](http://islandgirlintransit.com/) |
| 183 | [Jack and Jill Travel – World Travelers & Climbers](http://jackandjilltravel.com/) |
| 184 | [Jasmine Alley – Travel the World with a 9 to 5](https://www.jasminealley.com/) |
| 185 | [Jessie on a Journey – Taking you Beyond the Guidebook](http://jessieonajourney.com/) |
| 186 | [Jet Set Chick – Travel, Food & Lifestyle Blog](http://www.jetsetchick.com/) |
| 187 | [Jet Set Citizen – Interviews with Real People Pursuing Remarkable Lives](http://jetsetcitizen.com/) |
| 188 | [Joey L – Photos from the Road](http://joeyl.com/blog/) |
| 189 | [Johnny Africa – Expat Adventure Travel Blog](https://johnnyafrica.com/) |
| 190 | [Johnny Jet – Travel Deals](http://www.johnnyjet.com/) |
| 191 | [Jon is Travelling – The Light (and sometimes dark) Side of Travel](http://jonistravelling.com/) |
| 192 | [Jones Around the World – Global Adventures & Music Festivals](http://www.jonesaroundtheworld.com/) |
| 193 | [Journalist on the Run – Stories of Solo Adventures](http://journalistontherun.com/) |
| 194 | [Journey To Design – Travelling from a Designer’s Perspective](http://www.journeytodesign.com/) |
| 195 | [Journey Wonders – Culture & Adventure Travel Blog](https://www.journeywonders.com/) |
| 196 | [Journeys with Jessica – Travel Blog Focused on UK & European City Breaks](http://www.journeyswithjessica.net/) |
| 197 | [Joy and Journey – Female Expat Life and Exploration](http://joyandjourney.com/) |
| 198 | [Jungles In Paris – Stories of Nature & Culture](https://www.junglesinparis.com/) |
| 199 | [Just Go Places – Cultural, Luxury and Family Travel](http://www.justgoplacesblog.com/) |
| 200 | [Just One Way Ticket – A Travel and Lifestyle Blog](http://www.justonewayticket.com/) |
| 201 | [Just Travelous – A Bilingual Travel Blog](http://www.justtravelous.com/en/) |
| 202 | [Justin Goes Places – Solo Male Travel Blog](http://justingoesplaces.com/) |
| 203 | [Kaleidoscopic Wandering – Stories & Contemplations](http://kaleidoscopicwandering.com/) |
| 204 | [Kami and the Rest of the World – A Solo Female Off the Path](http://www.mywanderlust.pl/) |
| 205 | [Karolina Patryk – Couple Travel & All Inclusive Life](http://karolinapatryk.com/) |
| 206 | [Katie’s Postcards – Travel like a girl](https://www.katiespostcard.com/) |
| 207 | [Katrinka Abroad – Capturing Culture with Analogue Photography](http://katrinkaabroad.com/) |
| 208 | [Keep Calm & Travel – One Girl, One World, One Adventure.](http://www.keepcalmandtravel.com/) |
| 209 | [Ken Kaminesky – Travel Photography](http://blog.kenkaminesky.com/) |
| 210 | [Land Lopers – Exploring the World. One Adventure at a Time.](http://landlopers.com/) |
| 211 | [Lash World Tour – Cultural Insights, Travel Tips, and Adventure](http://www.lashworldtour.com/) |
| 212 | [Lavin Was Here – Female Backpacker and Adventure Travel](http://laviwashere.com/) |
| 213 | [Learning Escapes – Family Travel Blog](http://www.learningescapes.net/) |
| 214 | [Lee Abbamonte – The Youngest American to Visit All Countries in the World](http://leeabbamonte.com/) |
| 215 | [Legal Nomads – Sharing Travel Experiences through Food](http://legalnomads.com/) |
| 216 | [Letters To Barbara – A Travel Blog](https://letterstobarbara.com/) |
| 217 | [Live Like It’s The Weekend – Creative soul’s incurable case of wanderlust](http://www.livelikeitstheweekend.com/) |
| 218 | [Live Share Travel – Liberating luxury for the smart traveller](http://livesharetravel.com/) |
| 219 | [Live, Dream, Discover – Travel and Lifestyle Blog](http://www.livedreamdiscover.com/) |
| 220 | [Local Adventurer – Exploring New Cities](http://localadventurer.com/) |
| 221 | [Location 180 – Work from Anywhere](http://seanogle.com/) |
| 222 | [Longest Way Home – Going Beyond Travel](http://thelongestwayhome.com/blog/) |
| 223 | [Los Viajes de Paula – The Travels of Paula](https://losviajesdepaula.com/) |
| 224 | [Love & Road – We Travel, We Live, We Love](http://loveandroad.com/) |
| 225 | [Love Adventures – Dream Less, Adventure More](http://www.loveadventures.co.uk/) |
| 226 | [Love and London – Expat Life in London](http://loveandlondon.com/) |
| 227 | [Lovicarious – Discover Great Adventures](https://www.lovicarious.com/) |
| 228 | [Luggage Tags and Ticket Stubs – Travel & journalism adventures](http://luggagetagsandticketstubs.com/) |
| 229 | [Lulu Meets World – Fashion & Travel](http://www.lumenbeltran.com/) |
| 230 | [Luxury Backpack – Dedicated to Luxurious Travels on a Shoestring](http://luxurybackpack.com/) |
| 231 | [Maiden Voyage Travel – Travel for 20 Somethings](http://maiden-voyage-travel.com/) |
| 232 | [Make Time to See the World – Awesome Experiences for Time-Poor Travellers](http://www.maketimetoseetheworld.com/) |
| 233 | [Mallory on Travel – Making Every Day an Adventure](http://malloryontravel.com/) |
| 234 | [Man on the Lam – Shake the Shackles, Escape through Travel](http://manonthelam.com/) |
| 235 | [Man vs. Clock – Putting a Time Limit on Fear](http://manvsclock.com/) |
| 236 | [Mapping Megan – Adventure Travel Couple](http://www.mappingmegan.com/) |
| 237 | [Maps ‘n Bags – Travel tips & Inspiration from an International Couple](https://www.mapsnbags.com/) |
| 238 | [Maptia Travel Blog](https://maptia.com/) |
| 239 | [Maria Abroad – Culture, Food and Craft Beer](http://www.mariaabroad.com/) |
| 240 | [Matt Gibson – Outdoors Adventure Travel Writer & Photographer](http://matt-gibson.org/) |
| 241 | <http://www.matthew-woodward.com/> |
| 242 | [Microadventure Family – Travel Adventures In Your Backyard](http://microadventurefamily.com/) |
| 243 | [Midlife Milestones – Navigating Life’s Changes](http://midlifemilestones.com/) |
| 244 | [Midlife Road Trip – Food Travel Adventure](http://midliferoadtrip.tv/) |
| 245 | [Midnight Blue Elephant – Travel Stories, Luxury, Solo Travel](http://www.midnightblueelephant.com/) |
| 246 | [Migrationology – Ultimate Food Blog](https://migrationology.com/) |
| 247 | [Miles and Smiles Away – Travel Photography and Hotel Reviews](http://www.miles-smilesaway.com/) |
| 248 | [Mindful Travel by Sara – Travel & Photography Around the World](http://www.mindfultravelbysara.com/) |
| 249 | [Minority Nomad – Adventure & Culture Travel](http://minoritynomad.com/) |
| 250 | [Mint Notion – Travel and Personal Finance Blog Empowering Women](http://www.mintnotion.com/) |
| 251 | [Mom The Muse – A Family Travel Blog With a Humorous Twist](https://www.momthemuse.com/) |
| 252 | [Mountain Leon – A Travel, Camping, and Hiking Blog](https://mountainleon.com/) |
| 253 | [Multiculuriosity – Exploring Food Traditions Through Travel](http://www.multiculturiosity.com/) |
| 254 | [Muslim Travelers – Experience The World As A Traveler](http://www.muslimtravelers.com/) |
| 255 | [My Faces and Places – For Mature Travellers](http://myfacesandplaces.co.uk/) |
| 256 | [My Feet are Meant to Roam – Slow Travel and Responsible Tourism](http://myfeetaremeanttoroam.com/) |
| 257 | [My One Big Planet – Created to Inspire the Wanderlust in All of You to Travel](http://myonebigplanet.com/) |
| 258 | [My Peace Love Life Blog – A Hippie Lifestyle Blog About Travel & Fashion](http://mypeacelovelife.com/) |
| 259 | [My Tan Feet – Making Travel Fun & Worry Free](http://mytanfeet.com/) |
| 260 | [My Travel Affairs – Crazy Polish Girl Visiting One Country at a Time](http://www.mytravelaffairs.com/) |
| 261 | [Natpacker – Backpacking With Nat](https://natpacker.com/) |
| 262 | [Nattie on the Road – Travel Stories and Road Tested Tips](http://nattieontheroad.com/) |
| 263 | [Never Ending Footsteps – Traveller, Writer and Walking Disaster](http://neverendingfootsteps.com/) |
| 264 | [Never Ending Voyage – Life is Short and the World is Large](http://neverendingvoyage.com/) |
| 265 | [Nicole Buzzing – Lifestyle and Travel Blog from a Canadian Girl](http://www.nicolebuzzing.com/) |
| 266 | [Nomad Flag – Slow & Cultural Travel](https://nomadflag.com/) |
| 267 | [Nomad Revelations – Travel Blog & Adventures](http://www.joaoleitao.com/) |
| 268 | [Nomadic Boys – Gay couple travelling the world](http://www.nomadicboys.com/) |
| 269 | [Nomadic Matt](https://www.nomadicmatt.com/) |
| 270 | [Nomadic Samuel – Best of Every Place](http://nomadicsamuel.com/) |
| 271 | [Nomadical Sabbatical – A Nomad’s Musings on Long-Term Travel](http://nomadicalsabbatical.com/) |
| 272 | [Notes From The Road – Travel, Blogging and More](http://notesfromtheroad.com/) |
| 273 | [Off Path Travels – Inspiration, tips, and solid advice for your next adventure](https://www.offpathtravels.com/) |
| 274 | [Off The Beaten Track – Day Hikes & Round Trips](https://www.offthebeatentrack.se/) |
| 275 | [Off With The Kids – Family Travel Blog](http://offwiththekids.com/) |
| 276 | [Oh Darling, Let’s Be Adventurers – Travel Photography and Adventures](http://www.ohdarling.org/) |
| 277 | [On The Way Around – Adventure Travel Blog](https://onthewayaround.com/) |
| 278 | [Once in a Lifetime Journey – Online Destination for Luxury and Offbeat Travel](http://onceinalifetimejourney.com/) |
| 279 | [One Lucky Traveller– Bucket List Travel](http://oneluckytraveller.com/) |
| 280 | [One Step 4Ward – Dream Big, Travel Far, Live Full](http://onestep4ward.com/) |
| 281 | [One Way One World – Travel Guides, Photography & Fashion](http://www.onewayoneworld.com/) |
| 282 | [Oneika the traveller](http://www.oneika-the-traveller.com/) |
| 283 | [Ordinary Traveler – Adventure Travel Blog](http://ordinarytraveler.com/) |
| 284 | [Our Awesome Planet – Filipino Food Blog](http://www.ourawesomeplanet.com/) |
| 285 | [Out of Town Blog – Online Magazine with Travel News](http://outoftownblog.com/) |
| 286 | [Owl Over the World – Travel More & Worry Less](http://www.owlovertheworld.com/) |
| 287 | [Packs Light – Travel Young and Pack Light](http://packslight.com/) |
| 288 | [Paper Planes & Caramel Waffles – Travels for Those Who Love Beaches](http://www.paperplanesandcaramelwaffles.com/) |
| 289 | [Pauline Travels – Solo Female Travel Blogger](http://paulinetravels.com/) |
| 290 | [Pause The Moment – Travel from Around the World](http://pausethemoment.com/) |
| 291 | [PhilaTravelGirl – Affordable Luxury Solo Travel with a Side of Points](http://www.philatravelgirl.com/) |
| 292 | [Pinoy Adventurista – Adventure Blog of a Filipino Male](http://www.pinoyadventurista.com/) |
| 293 | [Points & Travel – Where Luxury Travel & Value Intersect](http://www.pointsandtravel.com/) |
| 294 | [Postcards from the World – Travel and Expat Life Blog](http://www.postcardsfromtheworld.com/) |
| 295 | [Practical Vagabonds– Exploring Life Outside the Box](https://practicalvagabonds.com/) |
| 296 | [Practical Wanderlust – Down to Earth Approach to Your Head in the Clouds](http://practicalwanderlust.com/) |
| 297 | [Pretravels – Your Journey Begins Here](http://www.pretravels.com/) |
| 298 | [Rachel Nicole – Mix of Fashion and Travel](http://www.rachelnicole.co.uk/) |
| 299 | [Real World Runaway – Professional Avoider of the Real World](http://realworldrunaway.com/) |
| 300 | [Renee Roaming – Let’s Get Lost](https://www.reneeroaming.com/) |
| 301 | [Rexy Edventures – Adventures of Handsome Backpacker](http://rexyedventures.com/) |
| 302 | [Roads & Kingdoms – Travel, Photography, Food](http://roadsandkingdoms.com/) |
| 303 | [Roaming Required – Traveling on Weekends & Short Breaks](http://roamingrequired.com/) |
| 304 | [Roaming the Americas – Responsible, Sustainable, Ethical Travel](http://roamingtheamericas.com/) |
| 305 | [Roar Loud – Adventure Travel Blog Based Out of New England](http://roarloud.net/) |
| 306 | [Rob Greenfield – Inspiring a Happy, Healthy Earth](http://robgreenfield.tv/) |
| 307 | [Rolling Fox – Resource for Camping and Hiking](http://rollingfox.com/) |
| 308 | [RTW Bound – Travel tips and stories from a full time traveller](http://rtwbound.com/) |
| 309 | [Runaway Brit – International Teacher Trying to Find Home](http://www.runawaybrit.com/) |
| 310 | [Sabrina Andrea Sachs – Minimalist and Meaningful Travels](http://sabrina-andrea-sachs.com/) |
| 311 | [Sailing with Totem – A Family’s Nomadic Journey](https://sv-totem.blogspot.com/) |
| 312 | [Salt In Our Hair – Awaken Your Wanderlust](https://www.saltinourhair.com/) |
| 313 | [Scrapbook Journeys -Exploring the Hidden Gems in Kenya and Beyond](http://www.scrapbookjourneys.com/) |
| 314 | [Screw the Average – Be An Outlier](https://screwtheaverage.com/) |
| 315 | [She Goes Global – Solo Mindful Travel for the Modern Woman](http://shegoesglobal.net/) |
| 316 | [Sidetracked Magazine](https://www.sidetracked.com/) |
| 317 | [Silks Road and Beyond – Adventures of Carly](http://carlysadventuresafar.com/) |
| 318 | [Silverspoon London – Luxury Travel & Food Blog](http://www.silverspoonlondon.co.uk/) |
| 319 | [Simply Travelled – Travel Tales](http://www.simplytravelled.com/) |
| 320 | [Six Year Gap Year – Solo and Long-Term Budget Travel](http://www.sixyeargapyear.com/) |
| 321 | [Skinny Backpacker – Step Into the Real World](http://skinnybackpacker.com/) |
| 322 | [Slower Travel – Whizzing around the UK on local buses](http://slowertravel.co.uk/) |
| 323 | [So Today We Found – A Travel Blog For Curious People](http://sotodaywefound.com/) |
| 324 | [Solitary Wanderer – Tales and Tips from Solo Female Traveler](http://www.solitarywanderer.com/) |
| 325 | [Solo Traveler Blog – Solo Tips and Stories](http://solotravelerblog.com/) |
| 326 | [Spanish And Go – Learn Spanish, Travel The World](https://spanishandgo.com/) |
| 327 | [Steph the Wayward Pilgrim – Wandering the World, One Dream at the Time](http://stephthewaywardpilgrim.com/) |
| 328 | [Sticky Mango Rice – An illustrated journey around the world](http://stickymangorice.com/) |
| 329 | [Stop Having a Boring Life – Travel Blog for People Chasing Dreams](http://stophavingaboringlife.com/) |
| 330 | [StreetTrotter – Travel & Lifestyle Touching a Meaning of Life](http://streettrotter.com/) |
| 331 | [Sugar and Stamps – A SF Based Travel and Food Blog](http://sugarandstamps.com/) |
| 332 | [Suitcase & Heels – Adventures of a Value-Conscious, Style-Minded Traveller](http://www.suitcaseandheels.com/) |
| 333 | [Sunset Travellers – Travelling the World One Sunset at a Time](http://sunsettravellers.com/) |
| 334 | [Sunshine Seeker – Budget Travel Guides & Tips](http://www.sunshineseeker.com/) |
| 335 | [Swedish Nomad – Every Day is An Adventure](https://www.swedishnomad.com/) |
| 336 | [Tales From a Fork – Travel and Lifestyle Blog for Foodies](http://www.talesfromafork.com/) |
| 337 | [Tapped Out Traveller – Exploring Europe with the Little Ones and Military Life](http://tappedouttravellers.com/) |
| 338 | [Teacake Travels – An Honest Solo Female Travel Blog](http://teacaketravels.com/) |
| 339 | [Teaching Traveling – Stories to Help Teachers Travel](http://www.teachingtraveling.com/) |
| 340 | [Tenacious Travel – Treks, cultural adventures and nomadic stories](http://www.http/tenacioustravel.com/) |
| 341 | [That Wanderlust – Travel Blog with Captivating Stories](http://thatwanderlust.com/) |
| 342 | [The Adventure Junkies – Don’t Dream It, Live It](http://www.theadventurejunkies.com/) |
| 343 | [The Adventure Travelers – RV, Sailing, and Hiking Blog](https://www.theadventuretravelers.com/) |
| 344 | [The Agape Co. – Adventure and budget travel](https://theagapecompany.com/) |
| 345 | [The Aussie Flashpacker – Aussie Girl & English Boy Travelling](http://www.theaussieflashpacker.com/) |
| 346 | [The Barefoot Nomad – Travel, Tech, Family & Fun](http://www.thebarefootnomad.com/) |
| 347 | [The Blog Abroad – Chronicles of an Adventure Junkie](http://theblogabroad.com/) |
| 348 | [The Blonde Abroad – Hopelessly in Love with the World.. and Bikinis!](https://theblondeabroad.com/) |
| 349 | [The Boho Chica – Cultures, Cuisines & Hiking Trails](http://thebohochica.com/) |
| 350 | [The Broke Backpacker – Traveling the World on the Cheap](http://www.thebrokebackpacker.com/) |
| 351 | [The Cheeky Traveler – Travel Like A King](http://the-cheekytraveler.com/) |
| 352 | [The Family Without Borders – Biggest European Family Travel Blog](http://thefamilywithoutborders.com/) |
| 353 | [The Fashion Matters – Source of Inspiration to Travel in Style](http://www.thefashionmatters.com/) |
| 354 | [The Fly Away Life – Encouraging Women to Pursue Their Passion](http://www.theflyawaylife.com/) |
| 355 | [The Girl with the Map Tattoo – Solo Female Travel and Expat Life](http://www.thegirlwiththemaptattoo.com/) |
| 356 | [The Insatiable Traveler – Travel Writing & Photography](https://theinsatiabletraveler.com/) |
| 357 | [The Intrepid Guide – Explore the World with Languages](https://www.theintrepidguide.com/) |
| 358 | [The Lost Backpack – Getting Lost is Half the Fun](http://thelostbackpack.com/) |
| 359 | [The Mind of Court – Life & Adventures of an Expat](http://themindofcourt.com/) |
| 360 | [The Mindful Mermaid – Insider Travel Tips & Living Mindfully](https://mindfulmermaid.com/) |
| 361 | [The Next Somewhere – Life’s a Journey](http://thenextsomewhere.com/) |
| 362 | [The Next Trip – Travel Tips & Destination Guides](https://www.thenexttrip.xyz/) |
| 363 | [The Passport Lifestyle – Photography for Travelers](http://www.thepassportlifestyle.com/) |
| 364 | [The Pink Backpack – Female Travel and Adventure Blog Featuring Photography](http://www.thepinkbackpack.com/) |
| 365 | [The Planet D – Adventure Travel Couple](http://theplanetd.com/) |
| 366 | [The Poor Traveler – Traveling at Any Cost](http://www.thepoortraveler.net/) |
| 367 | [The Radio Vagabond – Travel Podcast From Every Country In The World](http://www.theradiovagabond.com/) |
| 368 | [The Restless Worker – Part-Time Travel for Professional](http://www.therestlessworker.com/) |
| 369 | [The Roaming Street – A Young Female Traveller in Africa](http://www.theroamingstreet.com/) |
| 370 | [The Rover – Travel, People, and Culture](http://www.therover1.com/) |
| 371 | [The Savvy Backpacker – Europe on a Budget](http://thesavvybackpacker.com/) |
| 372 | [The Savvy Globetrotter – Travel Smarter](http://www.thesavvyglobetrotter.com/) |
| 373 | [The Shooting Star – A Girl Who Travels](http://the-shooting-star.com/) |
| 374 | [The Tales of a Traveler – Luxury Travel, Offbeat Locations and Road Trips](http://thetalesofatraveler.com/) |
| 375 | [The Thought Card – Make Travel a Financial Priority](http://www.thoughtcard.com/) |
| 376 | [The Toronto Seoulcialite – Life as an Expat in Korea](http://torontoseoulcialite.com/) |
| 377 | [The Travel Bite – Food Travel Fun](http://thetravelbite.com/) |
| 378 | [The Travel Episodes](http://en.travelepisodes.com/) |
| 379 | [The Travel Hack – Affordable Luxury & Weekend Breaks](http://thetravelhack.com/) |
| 380 | [The Travel Mamas – Explore the World with Kids](http://travelmamas.com/) |
| 381 | [The Travel Tart – Offbeat Tales of Travel Addict](http://thetraveltart.com/) |
| 382 | [The Traveling Squid – Traveling Awakens the Dreams in Us](http://thetravellingsquid.com/) |
| 383 | [The Traveller World Guide – Guide to Balancing Tequilla and Travel](http://thetravellerworldguide.com/) |
| 384 | [The Traveller’s Guide By #ljojlo – Aussie Couple Set to Embark on a Journey](http://www.hashtagljojlo.com/) |
| 385 | [The Travellina Hams – A family of 4 eating their way across the world](https://thetravellinghams.com/) |
| 386 | [The Trvl Blog – Budget Travel Blog](http://www.thetrvlblog.com/) |
| 387 | [The Wanderlust Effect – Inspiring Destinations for Global Citizens](http://www.thewanderlusteffect.com/) |
| 388 | [The Wayfarer’s Book – Beyond Paris, Miami and Bangkok](http://www.thewayfarersbook.com/) |
| 389 | [The World Incorporated – Travels Fueled by Weekends and Vacation Days](http://www.theworldincorporated.com/) |
| 390 | [The World Pursuit – Travel Different](http://theworldpursuit.com/) |
| 391 | [The World Travel Guy – Beautiful destinations, experiences, & hidden places](https://theworldtravelguy.com/) |
| 392 | [The Wrong Way Home – Long Term Travels and Local Culture](http://www.thewrongwayhome.com/) |
| 393 | [The Yacht Stew – Working On Yachts Around The World](https://theyachtstew.com/) |
| 394 | [There & Back Again – Extraordinary Travel for Extraordinary People](http://thereandbackagaintravel.com/) |
| 395 | [There She Goes Again – Slow, Stylish Travel](http://www.thereshegoesagain.org/) |
| 396 | [Think Elysian – Lifestyle Blog for Stylish Travelers](http://www.thinkelysian.com/) |
| 397 | [This Battered Suitcase – Travel Opinions, Fashion, and Lifestyle](http://www.thisbatteredsuitcase.com/) |
| 398 | [This FP Planet – Budget Traveller Trying to Escape the 9-5](http://www.thisfpplanet.com/) |
| 399 | [Thomas & Clay Get Away – Couples Adventure Travel](http://thomasandclaygetaway.com/) |
| 400 | [Thompson Woofed – The Travel Writings of Thompson Woofed](http://thompsonwoofed.com/) |
| 401 | [Time Travel Turtle – Go Beyond the Brouchure](http://www.timetravelturtle.com/) |
| 402 | [Tofu Traveler – Solo Travel and Finding Cultural Experiences](http://www.tofutraveler.com/) |
| 403 | [Trails Unblazed – Outdoor Adventures in Lesser Known Destinations](http://www.trailsunblazed.com/) |
| 404 | [Trans-Americas Journey – Road Tripping the Americas](http://trans-americas.com/) |
| 405 | [Trav Monkey – Travel Addict Living in London](http://www.travmonkey.com/) |
| 406 | [Travel and Keep Fit – Travel and Healthy Lifestyle Blog](http://travelandkeepfit.com/) |
| 407 | [Travel Babbo – Take Your Kids Everywhere](https://travelbabbo.com/) |
| 408 | [Travel Break – Connecting Travelers](http://www.travel-break.net/) |
| 409 | [Travel Dudes – For Travelers, By Travelers](http://www.traveldudes.org/) |
| 410 | [Travel Food Atlas – Interesting & delicious foods from around the world](http://www.travelfoodatlas.com/) |
| 411 | [Travel In Two Languages – Dual Language Travel Blog](http://travelintwolanguages.com/) |
| 412 | [Travel Is Life – Encouraging You To Travel More](https://travelislife.org/) |
| 413 | [Travel Junkie Julia – Journey of Adventurous Solo Female](http://www.traveljunkiejulia.com/) |
| 414 | [Travel Lemming – Let’s Travel the World!](https://travellemming.com/) |
| 415 | [Travel Made Simple – Overcome the Hurdles that Keep You from Traveling](http://travel-made-simple.com/) |
| 416 | [Travel Passionate – Around Greece & Beyond](http://travelpassionate.com/) |
| 417 | [Travel Squire – Travel Therapist](http://travelsquire.com/) |
| 418 | [Travel with Bender – Family Travel Blog](http://travelwithbender.com/) |
| 419 | [Travel With Bird – Our Travel Impressions](http://www.travelwithbird.com/) |
| 420 | [Travel with the Smile – Adventure Travel Blog Focusing on Outdoors](http://www.travelwiththesmile.com/) |
| 421 | [Travel World Notes – Travel tips for your next trip](http://travelworldnotes.blog/) |
| 422 | [Travel Yourself – A World Travellers Web Series](http://travelyourself.ca/1/) |
| 423 | [Travel, Books and Food – Female Solo Travel and Food Experiences](http://travelbooksfood.com/) |
| 424 | [Travel. Experience. Live. – Travel Experiences & Photography](http://www.travel-experience-live.com/) |
| 425 | [Traveling 9 to 5 – Get Out from Behind Your Desk](http://traveling9to5.com/) |
| 426 | [Traveling Colognian – Off the Beaten Path & Popular Places](http://travellingcolognian.com/) |
| 427 | [Traveling Honeybird – Eat Well, Travel Often, Drink Coffee](http://travelinghoneybird.com/) |
| 428 | [Traveling Weasels – German and British Couple Travel the World Slowly](http://www.travellingweasels.com/) |
| 429 | [Travellina – Hungarian Traveler & UNESCO Lover](https://travellina.hu/) |
| 430 | [Travelling King – Australian Blog on Travel & Finance](http://www.travellingking.com/) |
| 431 | [Travellous World – Stories About London and Travel](http://travellousworld.com/) |
| 432 | [Travels and Curiosities – Live smarter with this photo-driven travel blog](https://www.travelsandcuriosities.com/) |
| 433 | [Treksplorer – Smarter Urban Travel in Two Weeks or Less](http://www.treksplorer.com/) |
| 434 | [TripHackr – Travel Hacking for the Non-Travel Hacker](http://triphackr.com/) |
| 435 | [Two Bad Tourists – Peer-to-Peer Travel Tips & World Adventures](http://www.twobadtourists.com/) |
| 436 | [Two Birds Breaking Free – shoestring Budget Adventure Travel](http://www.twobirdsbreakingfree.com/) |
| 437 | [Two Find A Way – Young Couple’s Travel Adventure Through Life](https://twofindaway.com/) |
| 438 | [Two Monkeys Travel – Sustaining a Life of Travel](http://twomonkeystravelgroup.com/) |
| 439 | [Two Scots Abroad – Full-time Workers with a Life-Long Travel Habit](http://twoscotsabroad.com/) |
| 440 | [Typing To Taipei – Your One Stop Taipei Guide](https://typingtotaipei.com/) |
| 441 | [Un Poco de Sur – Adventure, Budget and Slow Travel](http://unpocodesur.com/) |
| 442 | [Uncharted Backpacker – Traveling To Uncharted Destinations](http://www.unchartedbackpacker.com/) |
| 443 | [Uncornered Market – Travel Wide, Live Deep](http://uncorneredmarket.com/) |
| 444 | [Universal Traveller – Luxury Adventure Travel](http://www.universal-traveller.com/) |
| 445 | [Unnavigated – Stories & Destination Guides For Travelers](http://unnavigated.com/) |
| 446 | [Untold Morsels – Travel Inspiration with Culture, Food and Family](http://www.untoldmorsels.com/) |
| 447 | [Vagabond3 – A Family Travel Blog for Adventure Lovers](http://vagabond3.com/) |
| 448 | [Vagrants of the World – Inspiration & Ideas for Lovers of Travel](http://www.vagrantsoftheworld.com/) |
| 449 | [Veronika’s Adventure – Best Adventures from Asia and Europe](http://www.veronikasadventure.com/) |
| 450 | [Voyage dy Vinec – Sailing the World with a Hen](http://voyagedyvinec.com/) |
| 451 | [Walk About Wanderer – Helping Budget Backpackers](http://walkaboutwanderer.com/) |
| 452 | [Walk Beside Me Blog – A Travel Couple Story](http://walkbesidemeblog.com/) |
| 453 | [Walkaboot – Adventure Travel Blog for Wild Hearted](http://www.walkaboot.ca/) |
| 454 | [Wander the Map – Adventure Travel Couple](http://wanderthemap.com/) |
| 455 | [Wander Your Way – Learning to Get Off the Beaten Path](http://wanderyourway.com/) |
| 456 | [Wanderer of the World – Inspiring the Next Generation of Female Wanderers](https://wandereroftheworld.co.uk/) |
| 457 | [Wandering On – Couple’s Adventure Travel](http://wanderingon.com/) |
| 458 | [Wandering Redhead – Traveling the World While Working as a Nurse](http://www.wanderingredhead.com/) |
| 459 | [Wandering Soul’s Wander Tales – Travel and Photography](https://ponderingmusings.blogspot.com/) |
| 460 | [Wandering Trader – Design Your Tomorrow](http://wanderingtrader.com/) |
| 461 | [WanderLuce – Travel Blog for Stylish Female Travelers](https://lucylucraft.com/) |
| 462 | [Wanderlust Chloe – Stylish Travel Blog for Solo Travelers](http://wanderlustchloe.com/) |
| 463 | [Wanderlust Logs – Caution is No Virtue in the Young](http://www.wanderlustlogs.com/) |
| 464 | [Wanderlust Storytellers – Family Travel Blog With Kids](https://www.wanderluststorytellers.com/) |
| 465 | [Wanderlusters – Live for Adventure](http://wanderlusters.com/) |
| 466 | [Wanderlustingk – Adventure and Off the Beaten Path Travel Guides](http://www.wanderlustingk.com/) |
| 467 | [Wayfaring Rachel – Travel, Fashion, Wellness for Bold Women](http://wayfaringrachel.com/) |
| 468 | [Wayfaring Views – Alternative Itineraries and Offbeat Adventures](https://wayfaringviews.com/) |
| 469 | [We Travel Around the World – Photos & Interviews from Around the World](http://www.wetravelaroundtheworld.com/) |
| 470 | [We’re The Russos – RV Living & Van Life](https://weretherussos.com/) |
| 471 | [What About Her – Personal Storytelling About Traveling](http://www.whatabouther.nl/) |
| 472 | [What Boundaries – Live Your Dream, Find Your Inspiration](http://www.whatboundariestravel.com/) |
| 473 | [What’s Dave Doing? – Living the Travel Dream](http://whatsdavedoing.com/) |
| 474 | [Where In Da World – Purposeful Adventure](https://whereindaworld.com/) |
| 475 | [Where In The World Is Rebecca? – Life as an Immigrant Away from Family](http://blog.beccajanestclair.com/) |
| 476 | [Where Is Czarina – Travel tips, guides, and budget traveling](https://www.whereisczarina.com/) |
| 477 | [Where Is Next – Travel, Food & Adventure While Holding a 9-5](http://whereisnext.com/) |
| 478 | [Where Is Tara – Affordable Luxury, Adventure, Art & Culture](http://whereistara.com/) |
| 479 | [While I’m Young and Skinny – Expat Life World Travel](http://whileimyoungandskinny.com/) |
| 480 | [While Out Riding – Exploring the World by Bicycle](http://whileoutriding.com/) |
| 481 | [While You Stay Home – There Is A World Out There](http://whileyoustayhome.com/) |
| 482 | [Who Needs Maps – Making Long Distance Relationship Work Through Travels](http://www.whoneedsmaps.com/) |
| 483 | [Why You Wander – Work smart, travel better](https://www.whyyouwander.com/) |
| 484 | [Wild About Travel – Travel Tales by Solo Addict](http://wild-about-travel.com/) |
| 485 | [Wild Junket – Travel Light, Travel Far](http://wildjunket.com/) |
| 486 | [Will Fly For Food – Exploring the World Through Food](http://www.willflyforfood.net/) |
| 487 | [Women On The Road – Solo Female Travel Resource](https://www.women-on-the-road.com/) |
| 488 | [Wonderful Wanderings – Travel Blog with Itineraries & Tips](https://wonderfulwanderings.com/) |
| 489 | World As I See It |
| 490 | [Woff the Beaten Path Sights and Budget Travels](http://worldinparis.com/) |
| 491 | [World Of Wanderlust – Home is Wherever you Rest your Head](http://www.worldofwanderlust.com/) |
| 492 | [World on a Whim – The Perfectionist’s Guide to Spontaneous Travel](http://worldonawhim.com/) |
| 493 | [World Travel Toucan – Travelling the world, one holiday at a time](https://worldtraveltoucan.com/) |
| 494 | [Y Round The World – Travel Experiences from 150 Countries](http://www.yroundtheworld.com/) |
| 495 | [Y Travel Blog – A Family Travel Blog](https://www.ytravelblog.com/) |
| 496 | [Yomadic – Wanderlust Commune](http://www.yomadic.com/) |
| 497 | [You Could Travel – Adventure Seekers and Japan Lovers](https://www.youcouldtravel.com/) |
| 498 | [Young Adventuress – Solo Female Travel Blog](http://youngadventuress.com/) |
| 499 | Young - Where to Next? |
| 500 | [Z Blonde Brunette Travel](http://blondebrunettetravel.com/) |

**Table S2.** 100 Spanish travel blogs analyzed by alphabetic order

| 1. [360 Travel](http://360travel.es/) |
| --- |
| 1. [A bordo del mundo](http://abordodelmundo.com/) |
| 1. [A tomar por mundo](https://atomarpormundo.com/) |
| 1. [Abuelita mochilera](https://abuelitamochilera.wordpress.com/) |
| 1. [Acróbata del camino](http://acrobatadelcamino.com/) |
| 1. [Ahorra y viaja](http://www.ahorrayviaja.com/) |
| 1. [Alan x el mundo](https://www.alanxelmundo.com/) |
| 1. [Algo que recordar](https://algoquerecordar.com/) |
| 1. [Aprendizaje viajero](https://www.aprendizajeviajero.com/) |
| 1. [Biciclown](http://www.biciclown.com/) |
| 1. [Biciklautak](http://biziklautak.com/) |
| 1. [Bike canine](http://www.bikecanine.com/) |
| 1. [Bitácoras de viaje](https://bitacorasdeviaje.com/) |
| 1. [Blog de IATI seguros](https://www.iatiseguros.com/blog/) |
| 1. [Blogtrip. El blog de viajes de Aristofennes](https://blogtrip.org/) |
| 1. [Camino salvaje](http://caminosalvaje.org/) |
| 1. [Cinecicleta](https://cinecicleta.wordpress.com/) |
| 1. [Colorado on the road](http://www.coloradoontheroad.com/) |
| 1. [Comiviajeros](https://comiviajeros.com/) |
| 1. [Con mochila](https://www.conmochila.com/) |
| 1. [Conocete viajando](http://conoceteviajando.com/) |
| 1. [Cronicas de una argonauta](https://cronicasargonauta.com/) |
| 1. [Dejarlo todo e irse](http://www.dejarlotodoeirse.com/) |
| 1. [Dos a la deriva](https://dosaladeriva.com/) |
| 1. [Dos mochilas en ruta](http://www.dosmochilasenruta.com/) |
| 1. [El blog de Paco Nadal](http://blogs.elpais.com/paco-nadal/) |
| 1. [El blog de viajes](https://www.elblogdeviajes.com/) |
| 1. [El mundo de pe a pa](http://elmundodepeapa.com/) |
| 1. [El pachinko](https://elpachinko.com/) |
| 1. [El próximo viaje](http://www.elproximoviaje.com/) |
| 1. [El rincón de Sele](http://www.elrincondesele.com/) |
| 1. [El turista](http://www.thetourist.tv/) |
| 1. [En el mundo perdido](http://www.enelmundoperdido.com/) |
| 1. [Familias en ruta](https://familiasenruta.com/) |
| 1. [Friki por viajar](https://www.frikiporviajar.com/) |
| 1. [Germen viajero](https://germenviajero.com/) |
| 1. [Guarida de secretos](http://www.guaridadesecretos.com/) |
| 1. [Guias Viajar](https://guias-viajar.com/) |
| 1. [Hey hey word](http://heyheyworld.com/) |
| 1. [Imanes de viaje](https://imanesdeviaje.com/) |
| 1. [Impulso viajero](https://impulsoviajero.com/) |
| 1. [Inteligencia viajera](https://inteligenciaviajera.com/) |
| 1. [Juan por el mundo](http://juanporelmundo.com/) |
| 1. [Juntos viajando](https://www.juntosviajando.com/) |
| 1. [Kombi rutera.](http://www.kombirutera.com.ar/) |
| 1. [Kris por el mundo](https://www.krisporelmundo.com/) |
| 1. [La gran escapada](https://www.lagranescapada.com/) |
| 1. [La maleta de Carla](https://lamaletadecarla.com/) |
| 1. [La viajera empedernida](http://www.laviajeraempedernida.com/) |
| 1. [La vida de viaje](https://lavidadeviaje.com/) |
| 1. [Lapiz nómada](https://www.lapiznomada.com/) |
| 1. [Las rutas de Laura](https://www.lasrutasdelaura.com/) |
| 1. [Los apuntes del viajero](https://www.losapuntesdelviajero.com/) |
| 1. [Los mundos de Celi](https://www.losmundosdeceli.com/) |
| 1. [Los traveleros](https://lostraveleros.com/) |
| 1. [Los viajes de Alba](https://losviajesdealba.com/) |
| 1. [Los viajes de Claudia](https://losviajesdeclaudia.com/) |
| 1. [Los viajes de Domi](https://losviajesdedomi.com/) |
| 1. [Los viajes de Moni](http://www.losviajesdemoni.com/) |
| 1. [Los viajes de Nena](http://losviajesdenena.com/) |
| 1. [Los viajes por el mundo](http://www.losviajesporelmundo.com/) |
| 1. [Lowcosteros](http://www.lowcosteros.com/) |
| 1. [Magia en el camino.](http://magiaenelcamino.com.ar/) |
| 1. [Marcando el polo](http://marcandoelpolo.com/) |
| 1. [Maruxaina y su mochila](http://maruxainaysumochila.com/) |
| 1. [Mi agenda viajera](https://miagendaviajera.com/) |
| 1. [Mi Aventura Viajando](http://miaventuraviajando.com/) |
| 1. [Mi mundo en la mochila](http://mimundoenlamochila.com/) |
| 1. [Mi viajar](http://miviajar.com/) |
| 1. [Mi vida en una mochila](http://www.mividaenunamochila.com/) |
| 1. [Mis viajes low cost](https://www.misviajeslowcost.com/) |
| 1. [Mochiadictos](https://www.mochiadictos.com/) |
| 1. [Mochileando por el Mundo](https://www.mochileandoporelmundo.com/) |
| 1. [Mochileros TV](https://mochilerostv.com/) |
| 1. [Mola viajar](https://www.molaviajar.com/) |
| 1. [Montañeros viajeros](http://www.montanerosviajeros.com/) |
| 1. [My guía de viaje](http://www.myguiadeviajes.com/) |
| 1. [Nomadarte](https://www.nomadarte.com/) |
| 1. [Nos vamos de rutica](http://nosvamosderutica.com/) |
| 1. [Objetivo viajar](http://www.objetivoviajar.com/) |
| 1. [Oliver Trip](http://olivertrip.com/) |
| 1. [Portal mochilero](https://www.portalmochilero.com/) |
| 1. [Road2help](https://road2help.org/) |
| 1. [Rodadas](https://www.rodadas.net/) |
| 1. [Rumbeando por ahí](http://www.rumbeandoporahi.com/) |
| 1. [Salta conmigo](https://saltaconmigo.com/blog/) |
| 1. [Sin parar de viajar](https://sinparardeviajar.com/) |
| 1. [Sinewan](http://www.sinewan.com/) |
| 1. [Sinmapa](http://www.sinmapa.net/) |
| 1. [Solo ida](http://soloida.com/) |
| 1. [Surfea tu vida](https://www.surfeatuvida.com/) |
| 1. [Tiempo de explorar](https://tiempodexplorar.com/) |
| 1. [Tiempo de mochilas](http://www.tiempodemochilas.com/) |
| 1. [Tragaviajes](https://www.tragaviajes.com/) |
| 1. [Trajinando por el mundo](http://trajinandoporelmundo.com/) |
| 1. [Un viaje de cuento](http://unviajedecuento.weebly.com/) |
| 1. [Una idea un viaje](http://unaideaunviaje.com/) |
| 1. [Vero4travel](https://www.vero4travel.com/) |
| 1. [Viaja por libre](https://www.viajaporlibre.com/) |
| 1. [Viajablog](https://www.viajablog.com/) |
